# Structural Characterization and Antioxidant Activity of β-Glucans from Highland Barley Obtained with Ultrasonic–Microwave-Assisted Extraction

**DOI:** 10.3390/molecules29030684

**Published:** 2024-02-01

**Authors:** Lihua Chen, Chunfeng Cui, Zhiheng Wang, Fuhong Che, Zhanxiu Chen, Shengbao Feng

**Affiliations:** 1 School of Perfume and Aroma Technology, Shanghai Institute of Technology, Shanghai 201418, China; cuicf728@163.com (C.C.); w17362631176@173.com (Z.W.); 2 Qinghai Huzhu Barley Wine Co., Ltd., Haidong 810500, China; chefuhong@qkj.com.cn (F.C.); chenzhanxiu@qkj.com.cn (Z.C.)

**Keywords:** highland barley, β-glucan, ultrasonic–microwave-assisted extraction, structural characterization, antioxidant activity

## Abstract

In order to efficiently extract β-glucan from highland barley (HBG) and study its structural characterization and antioxidant activity, ultrasonic–microwave-assisted extraction (UME) was optimized by the response surface method (RSM). Under the optimal extraction conditions of 25.05 mL/g liquid–solid ratio, 20 min ultrasonic time, and 480 W microwave intensity, the DPPH radical scavenging activity of HBG reached 25.67%. Two polysaccharide fractions were purified from HBG, namely HBG-1 and HBG-2. Structural characterization indicated that HBG-1 and HBG-2 had similar functional groups, glycosidic linkages, and linear and complex chain conformation. HBG-1 was mainly composed of glucose (98.97%), while HBG-2 primarily consisted of arabinose (38.23%), galactose (22.01%), and xylose (31.60%). The molecular weight of HBG-1 was much smaller than that of HBG-2. Both HBG-1 and HBG-2 exhibited concentration-dependent antioxidant activity, and HBG-1 was more active. This study provided insights into the efficient extraction of HBG and further investigated the structure and antioxidant activities of purified components HBG-1 and HBG-2. Meanwhile, the results of this study imply that HBG has the potential to be an antioxidant in foods and cosmetics.

## 1. Introduction

Highland barley is a main grain crop with economic prospects in the Qinghai–Tibet Plateau of China. Compared with other cereal crops, highland barley is rich in nutritional and functional compounds such as proteins [1], polyphenols [2], vitamins [3], unsaturated fatty acids [4], β-glucans [5], and so on [6]. As a natural biological macromolecule, β-glucan is generally regarded as one of the most crucial bioactive fractions in highland barley. Its tremendous potential has garnered extensive acknowledgement, as have its immunomodulatory activity [7], ability to reduce blood glucose levels [8], abilities in preventing and treating cardiovascular diseases [9], its antitumor effect [10], and its antioxidant activity [11]. β-glucan has antioxidant activity by scavenging radicals to terminate free radical-mediated oxidative stress. He et al. [12] found that the glucan had strong antioxidant activity and could be used as a promising antioxidant for food and pharmaceutical applications. At present, extensive research has established that the ability of β-glucan to scavenge radicals depends largely on its structure [13].

Commonly, the structure of polysaccharides is mainly influenced by various factors, including the source of raw materials and the methods employed for extraction [14]. Traditional methods of polysaccharide extraction include hot water extraction, alkali extraction, acid extraction, enzyme extraction, etc. Despite their advantages, they have problems related to long process times, low separation efficiency, reagent waste, high-energy consumption, and poor product quality, meaning that they cannot meet the current production needs [15]. To improve the extraction efficiency of polysaccharides while preserving their functional activity, a range of advanced extraction techniques, such as ultrasonic-assisted extraction, pulsed electric field extraction, high-hydrostatic-pressure processing, microwave-assisted extraction, and supercritical fluid extraction, have been developed [16]. It is worth noting that ultrasonic-assisted extraction has emerged as the most popular method for extracting natural substances from biological matrices. By employing cavitation, this advanced technique effectively releases a spectrum of bioactive compounds into the extraction solvent, thus facilitating their recovery [17]. Oprescu et al. [18] utilized ultrasonic-assisted extraction to extract rapeseed oil and compared it with the reflux method and impregnation method. The results showed that ultrasonic-assisted extraction had the advantages of a short extraction time and better extraction efficiency. Moreover, it was reported that ultrasonic-assisted extraction could improve the antioxidant activity of polysaccharides better than hot water extraction [19]. Microwave irradiation increased the dipole rotations, resulting in an increase in thermal energy in the reaction mixture, which improved the polysaccharide extraction efficiency [20].

Given these circumstances, RSM was selected to determine the optimal conditions of the ultrasonic–microwave-assisted extraction (UME) of HBG from the perspective of statistics. Subsequently, two polysaccharide fragments—HBG-1 and HBG-2—were purified from HBG, and their structural characteristics were studied based on their monosaccharide composition, molecular weight, chain conformation, UV, FT-IR, SEM, and ^1^H NMR spectrum analysis. Additionally, the antioxidant activities of HBG-1 and HBG-2 were evaluated through scavenging assays to DPPH radicals, ABTS radicals, hydroxyl radicals, and superoxide radicals. This study will contribute to the precise processing and utilization of HBG and demonstrate its potential to be a natural antioxidant in foods and cosmetics.

## 2. Results and Discussion

### 2.1. Single-Factor Test Results regarding Ultrasonic–Microwave-Assisted Extraction (UME)

**Figure 1:**
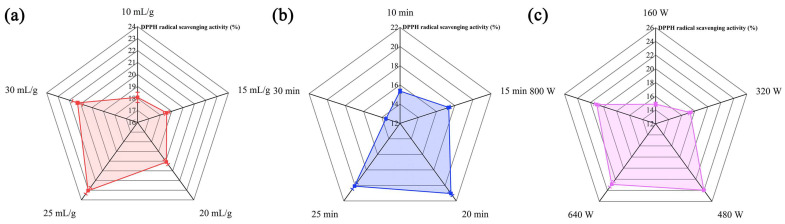
Effects of liquid–solid ratio (**a**), ultrasonic time (**b**), and microwave power (**c**) on the DPPH radical scavenging activity of HBG.

The UME of polysaccharides has been shown to lead to polysaccharides with better antioxidant activity [21], but polysaccharides can be affected by a series of operating parameters during the extraction process. Thus, selecting the appropriate major variables was crucial in enhancing antioxidant activity. The effect of liquid–solid ratio on DPPH radical scavenging activity is shown in Figure 1a. The DPPH radical scavenging activity of HBG displayed a noteworthy pattern of rapid growth before reaching a liquid–solid ratio of 25 mL/g. This could be attributed to the decreases in the concentration and viscosity of the entire extraction system as the solvent amount increased within this range, which facilitated the generation of an ultrasonic cavitation effect and enhanced the microwave’s mass transfer rate, leading to the disruption of the cell wall [22,23]. Consequently, polysaccharides were more likely to interact with DPPH radicals. However, at excessive liquid–solid ratios, the reduced substrate concentration might reduce mass transfer and the penetration of ultrasonic and microwave systems, leading to less cell wall destruction and a final reduction in the DPPH radical scavenging activity. Hence, it was concluded that the optimal liquid–solid ratio was 25 mL/g.

Figure 1b displays that the DPPH radical scavenging activity of HBG exhibited a progressive enhancement as the ultrasonic time was extended, reaching a maximum scavenging activity at 20 min. Prolonging the ultrasonic time beyond 20 min resulted in a decrease in scavenging activity. This might be attributed to the high shear and microjets generated by the acoustic cavitation effects of ultrasonication, which destroyed the polysaccharide structure, thus promoting more contact with DPPH radicals and improving scavenging activity. However, prolonged exposure to continuous cavitation could lead to polysaccharide degradation [24]. Such degradation can result in a decrease in DPPH radical scavenging activity after 20 min.

Figure 1c depicts that the DPPH radical scavenging activity of HBG kept an upward tendency as the microwave power intensity increased, reaching its peak at 480 W. The powerful penetrating ability and high-energy microwave radiation might explain this phenomenon. Microwave radiation had the ability to rapidly penetrate the interior of the cell, leading to efficient heating within a short duration. Additionally, this process resulted in the deep rupturing of the cell wall and enhanced the DPPH radical scavenging activity through mechanisms such as dipole rotation and ionic conductance [25]. However, due to the thermal instability of polysaccharides, strong microwave action would cause the degradation of the polysaccharides. Therefore, the optimal microwave power was determined to be 480 W.

### 2.2. RSM Optimization of Extraction Conditions

#### 2.2.1. Model Fitting and Statistical Analysis

Building on the results of our single-factor experimental analysis, we sought to explore the impact of the interaction between three independent variables: liquid–solid ratio, ultrasonic time, and microwave power. To investigate their combined effect on the DPPH radical scavenging activity of HBG, a series of 17 random sequence experiments were conducted. Multiple regression analysis was employed to establish a fitting formula that accurately describes the relationship between the response values and the factors:

(1) $$Y=24.98+0{.2312X}_{1}+2{.7X}_{2}+1{.19X}_{3}+0{.41X}_{1}X_{2}+1{.34X}_{1}X_{3}+0{.535X}_{2}X_{3}\;-\;3{{.14X}_{1}}^{2}\;-\;3{{.19X}_{2}}^{2}\;-\;3{{.77X}_{3}}^{2}$$

where Y is the DPPH radical scavenging activity of HBG; X_1_, X_2_, and X_3_ are the values for the liquid–solid ratio, ultrasonic time, and microwave power, respectively.

Table 1 presents the results of the analysis of variance (ANOVA) we carried out to determine the significance of each coefficient in the regression models. The order of influence for the three independent variables was X_3_ > X_2_ > X_1_, indicating that the influence of microwave power (X_3_) was the most significant. Additionally, the model reached a highly significant correlation (*p* < 0.0001), and the lack of fit of this model is insignificant (*p* > 0.05). The model R^2^ = 0.9853, R^2^_Adj_ = 0.9665, providing a more reliable measure of the model’s goodness of fit. Indeed, this regression model accurately reflected the relationship between the variable factors and the corresponding changes in response values.

#### 2.2.2. Response Surface Analysis

**Figure 2:**
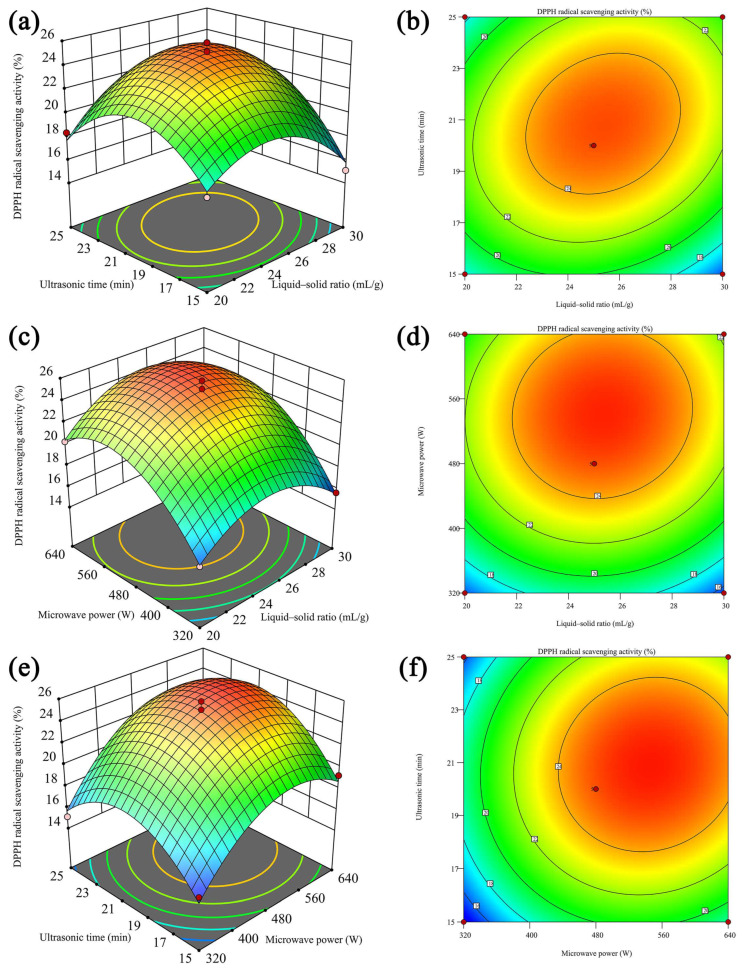
A 3D response surface plot and corresponding 2D contour plot of the interactive effects of liquid–solid ratio, ultrasonic time, and microwave power on the DPPH radical scavenging activity of polysaccharides. (**a**,**b**) are the interaction between liquid–solid ratio and ultrasonic time; (**c**,**d**) are the interaction between liquid–solid ratio and microwave power; (**e**,**f**) are the interaction between ultrasonic time and microwave power.

To visually illustrate the relationship between the interaction of each factor and the DPPH radical scavenging activity, 3D response surface plots, along with corresponding contour plots, were generated. Figure 2a,b demonstrate that the entire 3D surface exhibited a relatively steep profile, while the contour lines displayed an elliptical and dense shape. These characteristics suggested that both the liquid–solid ratio and ultrasonic time jointly influenced the DPPH radical scavenging activity of HBG. Moreover, the interaction between these two factors was found to be statistically significant (*p* < 0.05), highlighting their combined impact on the observed activity. The observed situation was also in agreement with the corresponding ANOVA results in Table 1. Figure 2c–f reveal that the interactions between liquid–solid ratio and microwave power and the interactions between ultrasonic time and microwave power were insignificant (*p* > 0.05, Table 1). As shown in Figure 2a,c,e, the downward openings observed in all 3D response surfaces served as clear indicators of the presence of extreme points within the regression model. Based on the comprehensive analysis conducted, the optimal HBG extraction conditions were determined to be as follows: liquid–solid ratio—25.05 mL/g; ultrasonic time—20.43 min; microwave power—488.85 W; and predicted DPPH radical scavenging activity—25.20%. After conducting practical operations, it was determined that the optimal ultrasonic time and microwave power for the extraction of HGB were 20 min and 480 W, respectively. Under the given conditions, the DPPH radical scavenging activity was determined to be 25.67%. This value closely aligned with the predicted values obtained from the regression equation, further affirming that the model designed by RSM had good adequacy and reliability and was suitable for the extraction of HBG. These findings highlight the effectiveness of this approach in maximizing the desired activity of HBG. Yang et al. [26] confirmed that UME could improve the biological activity of polysaccharides. Furthermore, when compared with HBG extracted by hot water extraction as reported previously [27], the optimum UME developed in this study demonstrated a clear advantage in enhancing antioxidant activity.

### 2.3. Separation and Purification Analysis of HBG

**Figure 3:**
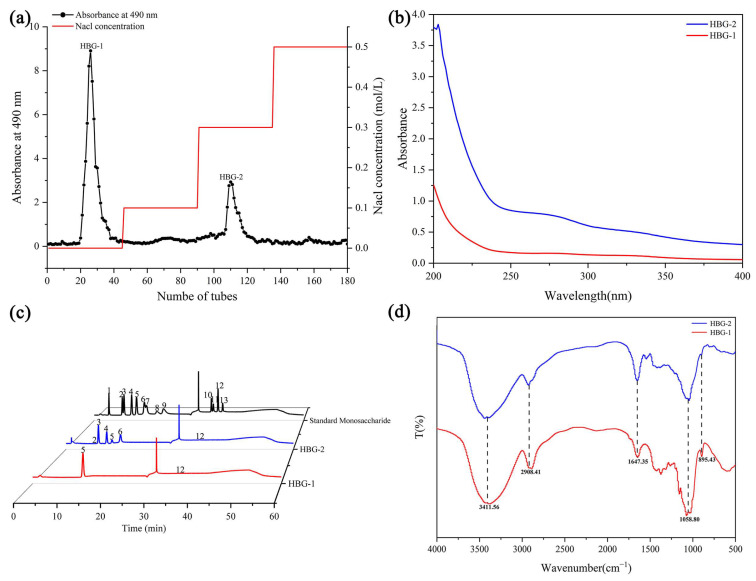
Fractionation and purification profiles of HBG-1 and HBG-2 on a DEAE-52 cellulose ion exchange column (**a**); UV spectrum scanning chart of HBG-1 and HBG-2 (**b**); monosaccharide composition maps for HBG-1 and HBG-2 (**c**) (1. fucose, 2. Rhamnose, 3. Arabinose, 4. Galactose, 5. Glucose, 6. Xylose, 7. mannose, 8. fructose, 9. ribose, 10. galacturonic acid, 11. Glucuronic acid, 12. mannuronic acid, 13. Guluronic acid) and FT-IR spectra of HBG-1 and HBG-2 (**d**).

To further clarify whether the composition of HBG was closely related to its antioxidant activity, we deproteinized the crude HBG and further purified it. As shown in Figure 3a, fractions with a relatively high content of HBG were obtained using different mobile phase concentrations (0, 0.1, 0.3, and 0.5 mol/L NaCl). For the two elution peaks with higher absorbance values, the eluates of the same composition were combined and named HBG-1 and HBG-2, respectively. Following our ultraviolet (UV) spectrum analysis, it was observed that the HBG-1 and HBG-2 solutions showed no UV absorption peaks at 260 nm or 280 nm (Figure 3b), indicating the absence of nucleic acids or proteins in these purified fractions.

### 2.4. Structural Characterization

#### 2.4.1. Monosaccharide Composition

Table 2 and Figure 3c show that there were differences in monosaccharide composition between HBG-1 and HBG-2. HBG-1 was composed of glucose (98.97%) and glucuronic acid (1.03%), while HBG-2 consisted of rhamnose (0.35%), arabinose (38.23%), galactose (22.01%), glucose (7.02%), xylose (31.60%), and glucuronic acid (0.80%). These results indicated that both polysaccharides were neutral heteropolysaccharides containing trace amounts of glucuronic acid [28]. Glucose was the dominant monosaccharide in HBG-1, while arabinose, galactose, and xylose were the primary monosaccharides in HBG-2. These findings indicated that HBG could be successfully separated into HBG-1 and HBG-2, and these fractions exhibited marked disparities in monosaccharide composition. Noticeable differences in particular composition and molar ratio between two polysaccharides could exert an influence on their structural characteristics and physicochemical properties, potentially giving rise to variations in antioxidant activity [29].

#### 2.4.2. Fourier-Transform Infrared Spectroscopy (FT-IR) Analysis

FT-IR spectroscopy has been extensively employed to elucidate the structural features of polysaccharides through the meticulous monitoring of the molecular vibrations within chemical bonds and functional groups [27]. It was observed that HBG-1 and HBG-2 had absorption peaks characteristic of polysaccharides (Figure 3d). The polysaccharide samples displayed a pronounced and broad absorption band in the range of 3500–3400 cm^−1^, indicating the presence of strong intermolecular hydrogen bonds or the stretching vibration of O–H groups [30]. The peak at 2908.41 cm^−1^ was ascribed to the asymmetric and symmetric stretching vibration of C–H. The presence of an absorption peak at 1647.35 cm^−1^ confirmed the existence of –COOH groups, indicating the presence of uronic acid in HBG-1 and HBG-2 [31]. The above findings corroborated our analysis of monosaccharide composition. The absorption peak observed at 1058.80 cm^−1^ could be attributed to the stretching vibration of C–O–C or C–O–H bonds, indicating that both HBG-1 and HBG-2 contain pyran rings [32]. However, the differences that emerged within the wavelength range of 1500–1000 cm^−1^ were primarily attributed to the diverse compositions and quantities of sugar bases in both polysaccharide samples. Additionally, the absorption peak at 895.43 cm^−1^ was characteristic of the absorption of the β-linked glycosidic bond [33]. Overall, both HBG-1 and HBG-2 exhibited similar structural characteristics.

#### 2.4.3. Molecular Weight and Chain Conformation

**Figure 4:**
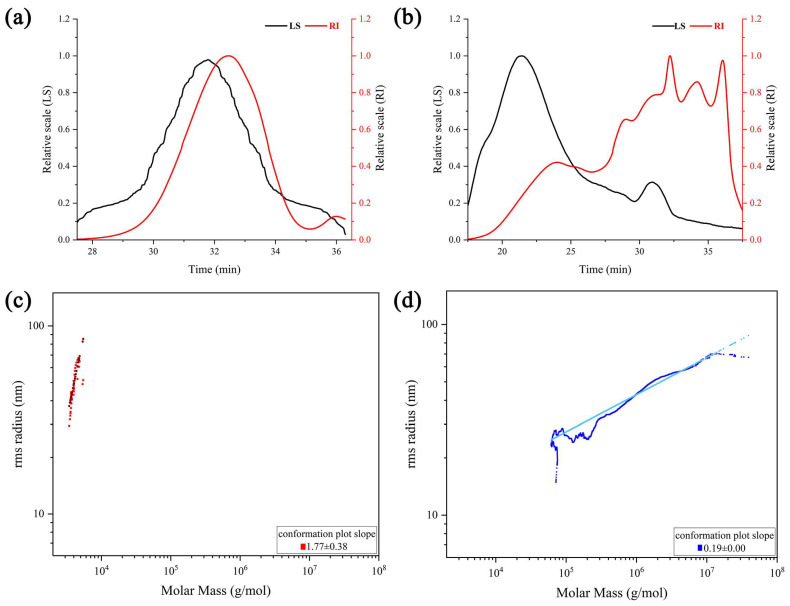
SEC-MALLS-RI chromatograms of HBG-1 (**a**) and HBG-2 (**b**) and the molecular conformations of HBG-1 (**c**) and HBG-2 (**d**).

The signals from differential RI and light scattering (LS) profiles of HBG-1 and HBG-2 are shown in Figure 4a,b. The LS profiles were sensitive to molecular weight, while RI provided insight into polysaccharide concentration [34]. The presence of a single and symmetrical peak in the RI profiles of HBG-1 suggested that the polysaccharide was homogeneous. Nevertheless, the RI profiles of HBG-2 exhibited multiple peaks within the retention time region, indicating the presence of aggregates and high-molecular-weight components. Furthermore, mainly one peak was detected by the LS detectors in HBG-1, seemingly belonging to one major constituent. The LS detectors in HBG-2 detected a large, broad peak alongside a smaller peak, which implied the presence of aggregates and higher-molecular-weight components [35].

The data regarding the weight-average (M_w_), number-average (M_n_), polydispersity index (M_w_/M_n_), and slope of molar mass against the molecular radius values of HBG-1 and HBG-2 are summarized in Table 2. The Mw value of HBG-1 was 5.24 kDa, which was considerably lower than that of HBG-2 (172.31 kDa). In contrast to HBG-2, the M_w_/M_n_ value of HBG-1 was relatively lower and close to 1, and the relatively restricted molecular weight distribution (low polydispersity) indicated excellent homogeneity, suggesting that it was a polysaccharide with a uniform composition and structure. Additionally, the chain conformations were determined by measuring the slope of molar mass against the molecular radius. The slope values obtained for HBG-1 and HBG-2 were 1.77 ± 0.38 and 0.19 ± 0.00, respectively. Figure 4c illustrates that HBG-1 exhibited a rigid rod-like conformation, hinting at its potential existence in water as a linear structure composed of a triple-helix conformation. Compared with HBG-1, the chain conformation of HBG-2 underwent a transition from a hollow sphere state to a solid sphere state (Figure 4d), resulting in an increase in molecular weight, which was consistent with their molecular weight results.

#### 2.4.4. Microstructure

**Figure 5:**
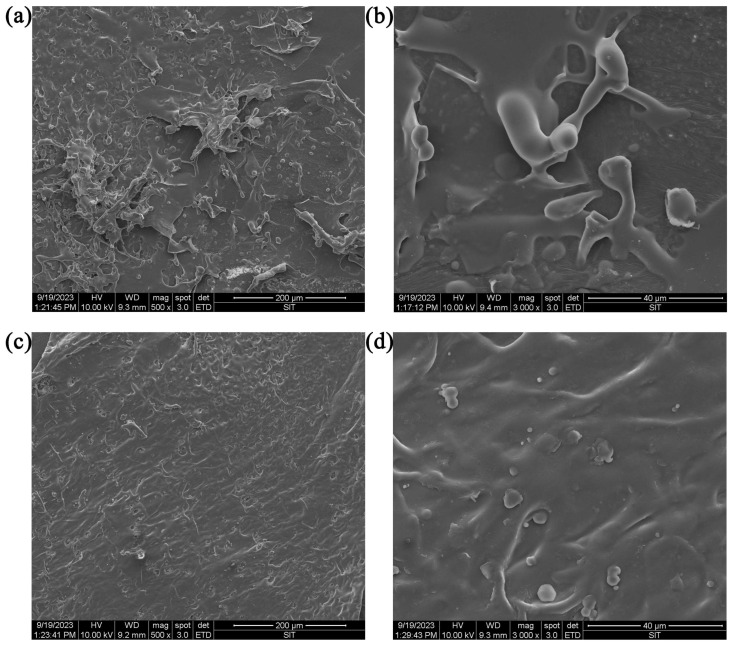
SEM images of HBG-1 ((**a**), 500×; (**b**), 3000×) and HBG-2 ((**c**), 500×; (**d**), 3000×).

Scanning electron microscope (SEM) images (taken under a magnification of 500 and 3000) of HBG-1 and HBG-2 are presented in Figure 5. It can be seen that HBG-1 possessed a smooth strip and flake structure which could potentially be attributed to the robust interaction between molecular chains [36]. Furthermore, numerous pores were observed on the surface of HBG-1. The HBG-2 component was massive, with debris on the surface, an uneven size and distribution, and holes in its structure. Generally, the fragmentation degree of HBG-1 was greater than that of HBG-2. Through the integration of monosaccharide composition analysis and molecular weight determination, we found that the greater the fragmentation degree, the smaller the molecular weight. Accordingly, the differences in microstructure could be attributed to the diversities in molecular weight, monosaccharide composition, and chain conformation [37].

#### 2.4.5. Analysis of the ^1^H Nuclear Magnetic Resonance (NMR) Spectra

**Figure 6:**
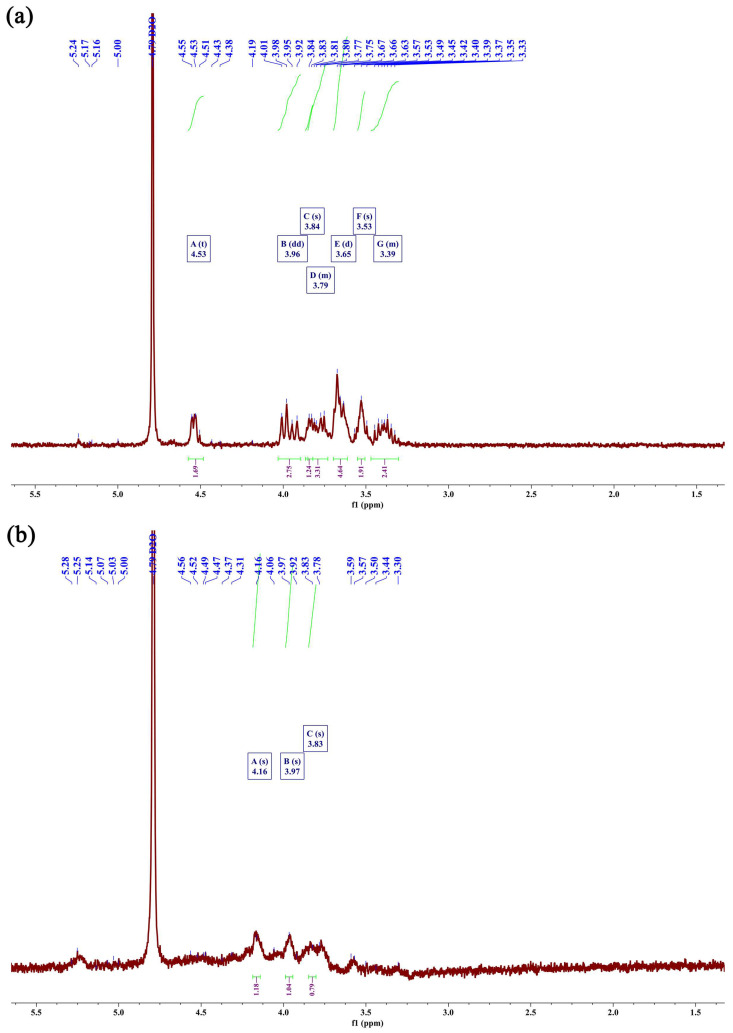
^1^H NMR spectra of HBG-1 (**a**) and HBG-2 (**b**).

To further investigate the structures of HBG-1 and HBG-2, the two polysaccharides were analyzed by ^1^H NMR (Figure 6). In general, the ^1^H NMR signals of the polysaccharides were confined to a relatively narrow range, posing challenges in identifying the spin system. However, the information obtained from the ^1^H NMR spectra was helpful in determining the configuration of glycosidic bonds and identifying specific residues or groups within the intricate structure of polysaccharides [38]. As shown in Figure 6, the prominent signal observed at 4.79 ppm, which served as a reference peak, was attributed to the presence of D_2_O. The main chemical shifts were observed between 3.30 and 5.30 ppm, representing typical polysaccharide signals. The major anomeric hydrogens signals of HBG-1 and HBG-2 appeared between 4.30 and 4.60 ppm, emphasizing the presence of the β-glucan form [39]. Notably, the signals detected in the anomeric hydrogen region might not exclusively originate from anomeric hydrogen alone. Moreover, 3.50–3.80 ppm indicated methoxy proton peaks of HBG-1 and HBG-2, respectively [40]. In summary, the insights gleaned from the aforementioned ^1^H NMR spectra indicated that both the HBG-1 and HBG-2 obtained in this study were characteristic spectra of β-glycosidically linked pyranose, aligning with the findings of the FT-IR analysis.

### 2.5. Antioxidant Activities of HBG-1 and HBG-2

#### 2.5.1. DPPH Radical Scavenging Activity

**Figure 7:**
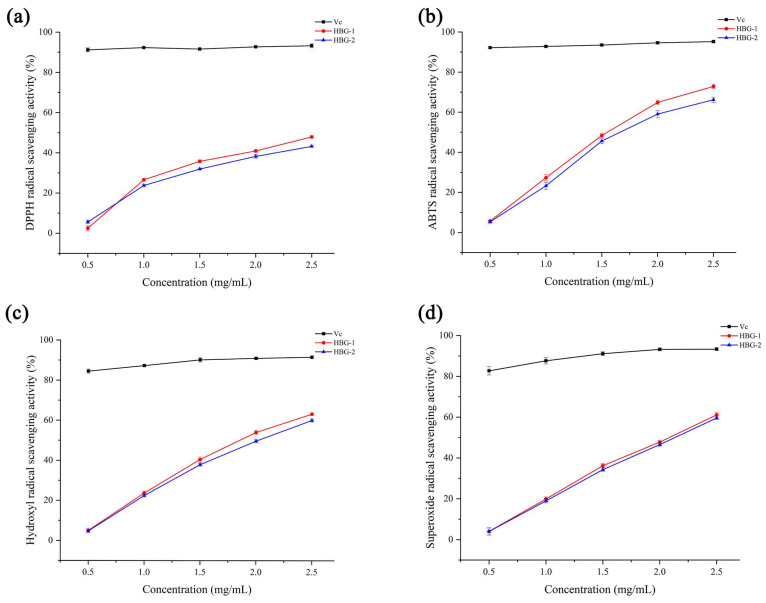
The scavenging activities of HBG-1 and HBG-2 on DPPH radicals (**a**), ABTS radicals (**b**), hydroxyl radicals (**c**), and superoxide radicals (**d**).

The antioxidant mechanism of DPPH radical scavenging was due to the capture of other radicals by unpaired electrons, changing the color of DPPH from purple to colorless [41,42]. As depicted in Figure 7a, both HBG-1 and HBG-2 exhibited concentration-dependent DPPH radical scavenging activity within the range of 0.5 to 2.5 mg/mL. The maximum scavenging activities of HBG-1, HBG-2, and Vc were 47.89 ± 0.94%, 43.19 ± 0.56%, and 93.20 ± 0.63%, respectively, at the level of 2.5 mg/mL. In contrast, the scavenging activity of HBG-1 was superior to that of HBG-2 due to it having a smaller molecular weight and a larger surface area, making it more convenient for interactions with DPPH radicals.

#### 2.5.2. ABTS Radical Scavenging Activity

ABTS radicals reacted with potassium persulfate to form a stable blue–green complex which was decolorized by antioxidants and used to evaluate antioxidant capacity [43]. HBG-1 exhibited higher ABTS radical scavenging activities compared to HBG-2, which could potentially be attributed to its elevated uronic acid content (Figure 7b). The active hydroxyl groups derived from the uronic acids were likely to exert a crucial influence on ABTS radical scavenging [44]. As their concentration increased, the ABTS radical scavenging activities of them showed a corresponding rise before reaching relatively stable values. At 2.5 mg/mL, the scavenging activities of HBG-1, HBG-2, and Vc were 72.87 ± 1.04%, 66.22 ± 1.29%, and 95.20 ± 0.42%, respectively. These results provide evidence to support the notion that both polysaccharides possessed remarkable antioxidant capabilities within hydrophilic systems.

#### 2.5.3. Hydroxyl Radical Scavenging Activity

The hydroxyl radical, with its highly reactive nature, has the capacity to break down macromolecules within biological systems [45]. HBG-1 and HBG-2 exhibited certain scavenging activities for the hydroxyl radical. However, their effectiveness was found to be lower than that of Vc (Figure 7c). As the concentration of both polysaccharides increased, a notable enhancement in the hydroxyl radical scavenging capacity was observed. Upon reaching a concentration of 2.5 mg/mL, the maximum scavenging values for HBG-1, HBG-2, and Vc were 63.19 ± 0.67%, 60.02 ± 0.77%, and 91.6 ± 0.74%, respectively. The hydroxyl radical scavenging activity of both polysaccharides mainly came from the dissociation of the C–H bond. The hydroxyl radical scavenging effect of HBG-1 was more significant than that of HBG-2, which could potentially be attributed to the fact that HBG-1 supplied a greater amount of hydrogen to the hydroxyl radicals, resulting in the production of stable radicals that impede the chain reaction [22].

#### 2.5.4. Superoxide Radical Scavenging Activity

Superoxide radicals are a highly toxic reactive oxygen species which cause severe damage to tissues or organs [46]. The results regarding the superoxide radical scavenging activities of HBG-1, HBG-2, and Vc are shown in Figure 7d. Comparatively, the superoxide radical scavenging activity of Vc was greater than that of HBG-1 and HBG-2. Both polysaccharides showed a radical scavenging ability in a concentration-dependent manner. Moreover, at 2.5 mg/mL, the scavenging activities of HBG-1, HBG-2, and Vc were 61.12 ± 1.26%, 59.60 ± 0.82%, and 93.30 ± 0.72%, respectively. The results indicated that both HBG-1 and HBG-2 possessed effective capabilities for scavenging superoxide radicals.

Collating the results of this study, it is evident that the antioxidant activities of HBG-1 and HBG-2 followed the following order: ABTS radicals > hydroxyl radicals > superoxide radicals > DPPH radicals. A multitude of factors, encompassing molecular weight, uronic acid, branching rate, and β-glycosidic linkages, played a predominant role in shaping the antioxidative properties of polysaccharides due to the differences in the sources of the raw materials and extraction methods [47,48]. Both polysaccharide fractions showed excellent antioxidant capacities, which might be related to the use of UME. Zhang et al. [25] compared UME with ultrasonic-assisted extraction, microwave-assisted extraction, and hot water-assisted extraction. The results confirmed that UME enhanced the antioxidant activity of *Dictyophora indusiata* polysaccharides. Compared with low-molecular-weight polysaccharides, the tighter structure resulted in a limited formation of hydroxyl groups in virtue of the stronger interaction of intramolecular hydrogen bonds in high-molecular-weight polysaccharides. The electrophilic ketone or aldehyde group within uronic acid enhanced the reactivity of the hydrogen atom, promoting its liberation from the hydrogen–oxygen bonds and ultimately contributing to the attainment of antioxidant effects. Therefore, the content of uronic acid exerted a certain influence on the antioxidant activity of the polysaccharides. The variances in structure between HBG-1 and HBG-2 might account for the superior antioxidant activity of HBG-1, which is potentially due to its lower molecular weight and elevated uronic acid content. Nevertheless, given the intricate nature of antioxidant mechanisms, further exploration was required to elucidate the specific correlation between antioxidant activity and polysaccharide structure.

## 3. Materials and Methods

### 3.1. Materials

Highland barley was provided by Qinghai Huzhu Barley Wine Co., Ltd. (Haidong, China). DEAE-52 cellulose was obtained from Beijing Solarbio Science & Technology Co., Ltd. (Beijing, China). Monosaccharide standards and D_2_O (D, 99.9%) were gained from Sigma-Aldrich Co. (St. Louis, MO, USA). All the reagents used were of analytical grade.

### 3.2. UME of HBG

#### 3.2.1. Extraction of Crude Polysaccharides

To achieve a consistent powder, the dried highland barley was carefully smashed and then sifted through a 60-mesh sieve prior to the extraction process. Subsequently, the powder underwent a pre-extraction process using 80% ethanol at 80 °C for 4 h at a liquid–solid ratio of 10 mL/g to eliminate pigments, fat, and other impurities soluble in alcohol. After the organic solvent was volatilized, the residues insoluble in ethanol were collected and dried for further operation. The UME of HBG followed a previously reported method with appropriate modifications [49]. In short, the pretreated powder was combined with deionized water at various liquid–solid ratios (10–30 mL/g). The mixture was then subjected to sonication using an ultrasonic power of 480 W for different durations (10–30 min). After that, the mixture was subjected to microwave heating for 4 min at different microwave powers (160–800 W). The samples were extracted with deionized water in a 55 °C water bath for 2 h, and the supernatants were centrifugated and collected. The supernatants were subsequently concentrated to 1/3 of their original volume using a vacuum rotary evaporator. After concentrating the solution, a precipitation process was carried out using 95% ethanol at 4 °C for 12 h. The resulting mixture was then centrifugated and retained for precipitation, redissolved in deionized water, and subsequently subjected to dialysis (above 3.5 kDa) for 48 h. The retention solution was freeze-dried to obtain the crude HBG. The DPPH radical scavenging activity of the crude HBG was evaluated using a previously described method [50]. Briefly, a mixture of 2 mL of HBG samples (1 mg/mL) and 2 mL DPPH solution (0.2 mmol/L) were incubated in dark conditions for 30 min. Then, the absorbance was determined at 517 nm as A_i_. For the blank control (A_j_), a solution consisting of 95% ethanol was utilized in place of the DPPH solution. Similarly, a control solution (A_c_) was prepared by substituting the sample solution with a 95% ethanol solution. The formula used to determine the DPPH radical scavenging activity is given below:

(2) $$\mathrm{DPPH}\;\mathrm{radical}\;\mathrm{scavenging}\;\mathrm{activity}\;(\%)=\left(1\;-\;\frac{A_{i}{\;-\;A}_{j}}{A_{c}}\right)\;\times \;100$$

#### 3.2.2. Single-Factor Experiments

Successively, the effects of three single factors, namely liquid–solid ratio, ultrasonic time, and microwave power, were meticulously examined in the UME of HBG. Each experiment was dedicated to altering a single factor while maintaining unwavering constancy in the remaining factors. For the single-factor experiments, the liquid–solid ratio was tested at different levels: 10 mL/g, 15 mL/g, 20 mL/g, 25 mL/g, and 30 mL/g. The ultrasonic time was varied, and the following ultrasonic times were used: 10 min, 15 min, 20 min, 25 min, and 30 min. The microwave power values used for the experiments were 160 W, 320 W, 480 W, 640 W, and 800 W.

#### 3.2.3. Response Surface Optimization

Given the outcomes of the single-factor analysis, RSM was employed to identify the optimal conditions for the UME of HBG. Table 3 displays the Box–Behnken design, which utilized the DPPH radical scavenging activity of HBG as the response value. The liquid–solid ratio (X_1_), ultrasonic time (X_2_), and microwave power (X_3_) were selected as the three factors in the design. Each factor was assigned three appropriate levels to create a three-factor and three-level response surface test. To predict the optimal point, the regression fitting model was represented by the following second-order polynomial:

(3) $${Y=\beta }_{0}+\sum \beta_{i}X_{i}+\sum \beta_{\mathrm{ii}}{X_{i}}^{2}+\sum \sum \beta_{\mathrm{ij}}X_{i}X_{j}$$

where Y is the dependent variable; X_i_, X_j_ are the independent variables representing liquid–solid ratio, ultrasonic time, and microwave power, respectively. β_0_ is a constant, and β_i_, β_ii_, and β_ij_ are the coefficients estimated by the model.

### 3.3. Purification of HBG

The deproteinization of crude HBG was conducted using Sevag reagent in a ratio of 1:4 (n-butanol: chloroform). To further isolate pure HBG, a 20 mg/mL solution of crude HBG was subjected to separation using a DEAE-52 cellulose ion exchange column (φ 2.5 cm × 60 cm). After the HBG solution was loaded, the column with HBG solution was eluted with a stepwise gradient of 0, 0.1, 0.3, and 0.5 mol/L sodium chloride (NaCl) solution at a flow rate of 1 mL/min, respectively. The eluent was collected in a test tube (10 mL/tube), and the polysaccharide concentration was detected by the phenol–sulfuric acid method to draw an elution curve. Two portions were eluted from the HBG solution, and the obtained eluents were pooled and dialyzed (above 3.5 kDa) for 48 h. Two purified fractions—HBG-1 and HBG-2—were obtained after the freeze-drying of the retention solution.

### 3.4. Structural Characterization of HBG-1 and HBG-2

#### 3.4.1. UV Spectroscopic

HBG-1 and HBG-2 were prepared into a 1 mg/mL solution. The UV-vis spectra of the samples were scanned using a Shimadzu UV-1900 spectrophotometer (Shimadzu Corporation, Kyoto, Japan), covering a wavelength range of 200–400 nm.

#### 3.4.2. FT-IR

The dried HBG-1 and HBG-2 were ground uniformly with KBr and pressed into a 1 mm slice. The peak spectra were acquired using a Nicolet iS20 FT-IR spectrometer between 4000 and 400 cm^−1^.

#### 3.4.3. Monosaccharide Composition

The monosaccharide compositions of HBG-1 and HBG-2 were investigated by high-performance anion-exchange chromatography (HPAEC) [51]. Briefly, HBG-1 and HBG-2 samples (5 mg) were dissolved with 1 mL of trifluoroacetic acid (2 mol/L), hydrolyzed at 121 °C for 2 h in a sealed tube, and then washed repeatedly with methanol under a nitrogen stream. The residue was redissolved in deionized water and then filtered using a microporous filtering film with a pore size of 0.22 μm. Subsequently, the sample extracts were analyzed by anion-exchange chromatography (Thermo Fisher, Waltham, MA, USA, Dionex ICS 5000+ chromatography system) using a pulsed amperometric detector equipped with a CarboPac PA20 anion-exchange column (3 mm × 150 mm). The mobile phase A was H_2_O, and the mobile phase B was composed of 0.1 mmol/L NaOH and 0.2 mmol/L NaAc. The rate of mobile phase was 0.5 mL/min, and the sample injection volume was 5 μL. The calibration curve was obtained via the analysis of monosaccharide standards.

#### 3.4.4. SEC-MALLS-RI Measurement

For the size-exclusion chromatography–multiangle laser light scattering-refractive index (SEC-MALLS-RI), a DAWN HELEOS-II laser photometer (Wyatt Technology Co., Santa Barbara, CA, USA) equipped with three tandem columns (300 × 8 mm, Shodex OH-pak SB-805 and 803, Showa Denko K.K., Tokyo, Japan) was utilized to measure the molecular weights and chain conformations of HBG-1 and HBG-2 [52]. HBG-1 and HBG-2 were dissolved in 0.1 mol/L NaNO_3_ aqueous solution containing 0.02% NaN_3_ at the concentration of 1 mg/mL and filtered through a filter of 0.45 μm filter. The flow rate was 0.6 mL/min, and 100 μL of sample was injected. The dn/dc value of the fractions in 0.1 mol/L NaNO_3_ aqueous solution containing 0.02% NaN_3_ was determined to be 0.141 mL/g.

#### 3.4.5. SEM

To facilitate conductivity, the dried HBG-1 and HBG-2 fractions were fixed on a conductive metal holder and coated with a thin layer of gold film. The SEM images were captured by SEM (FEI Quanta 200 FEG, Thermo Scientific, Waltham, MA, USA) at the magnifications of 500× and 3000×, respectively.

#### 3.4.6. NMR

A solution of HBG-1 and HBG-2 was delicately formulated at a concentration of 20 mg/mL utilizing D_2_O as the solvent. The Qone AS400 NMR spectrometer (Q.One Instruments Ltd., Wuhan, China) was used to determine the ^1^H NMR spectra.

### 3.5. In Vitro Antioxidant Activity Assays

The DPPH radical, ABTS radical, hydroxyl radical, and superoxide radical scavenging activities of HBG-1 and HBG-2 were determined using previously described methods [53,54,55,56]. The detailed process is available in the Supplementary Materials.

### 3.6. Statistical Analyses

Each experiment was repeated three times, and the data obtained are presented as means ± standard deviation. Statistical analyses were conducted using SPSS 18.0 and Origin Pro 2021 (Origin Lab Corporation, Northampton, MA, USA). The data were analyzed using ANOVA and Duncan’s multiple range tests.

## 4. Conclusions

In the current study, the UME technique demonstrated remarkable efficiency in extracting HBG. Through RSM analysis, the optimal extraction conditions for HBG were successfully determined. Two polysaccharide fractions—HBG-1 and HBG-2—were purified from HBG. Structural characterization revealed that they shared similar but not identical characteristics. Our determination of radical scavenging activity showed that HBG-1 had a smaller molecular weight and exhibited better antioxidant activity. These findings provide evidence that the antioxidant activity of polysaccharides is intricately linked to their unique structural characteristics and molecular weight. However, in order to fully unravel the intricate structure–activity relationship, further research and exploration was deemed necessary. Overall, this study not only provides new insights into the efficient extraction of HBG but also reveals the tremendous potential of HBG-1 and HBG-2, purified from HBG, as natural antioxidants.

## Figures and Tables

**Table 1:** ANOVA for the regression response surface model.

Source	Sum of Squares	DF	Mean Square	F-Value	*p*-Value	*p*
Model	249.33	9	27.70	52.29	<0.0001	significant
X_1_	0.4278	1	0.4278	0.8074	0.3987	
X_2_	11.35	1	11.35	21.43	0.0024	
X_3_	58.32	1	58.32	110.07	<0.0001	
X_1_X_2_	7.16	1	7.16	13.51	0.0079	
X_1_X_3_	0.6724	1	0.6724	1.27	0.2971	
X_2_X_3_	1.14	1	1.14	2.16	0.1850	
X_1_^2^	41.48	1	41.48	78.29	<0.0001	
X_2_^2^	59.80	1	59.80	112.87	<0.0001	
X_3_^2^	51.17	1	51.17	96.58	<0.0001	
Residual	3.71	1	0.5298			
Lack of fit	2.55	1	0.8541	2.95	0.1616	Not significant
Pure Error	1.15	4	0.2886			
Cor Total	253.04	16				
R^2^	0.9853					
R^2^_Adj_	0.9665					

**Table 2:** The physicochemical properties of HBG-1 and HBG-2.

Sample	HBG-1	HBG-2
M_w_ (kDa)	5.24	172.31
M_n_ (kDa)	4.94	37.31
M_w_/M_n_	1.06	4.62
Slope	1.77 ± 0.38	0.19 ± 0.00
Monosaccharide (mol%)		
Rhamnose	-	0.35
Arabinose	-	38.23
Galactose	-	22.01
Glucose	98.97	7.02
Xylose	-	31.60
Glucuronic acid	1.03	0.80

**Table 3:** Experimental design and results derived from the Box–Behnken design.

Run	Liquid–Solid Ratio (X_1_, mL/g)	Ultrasonic Time (X_2_, min)	Microwave Power (X_3_, W)	DPPH Radical Scavenging Activity (Y, %)
1	25	20	480	25.86
2	25	20	480	24.68
3	20	20	640	20.28
4	30	25	480	21.26
5	25	15	320	14.91
6	25	20	480	24.64
7	25	15	640	19.26
8	20	20	320	15.72
9	20	25	480	18.37
10	30	20	640	21.81
11	25	20	480	24.59
12	20	15	480	17.56
13	25	20	480	25.13
14	30	20	320	15.61
15	30	15	480	15.10
16	25	25	640	21.61
17	25	25	320	15.12

## Data Availability

Data are contained within the article and Supplementary Materials.

## Supplementary Materials

The following supporting information can be downloaded at: https://www.mdpi.com/article/10.3390/molecules29030684/s1. The detailed determination process of ABTS radical, hydroxyl radical and superoxide radical scavenging activity.
