# Supplementary material for: Structural Characterization and Antioxidant Activity of β-Glucans from Highland Barley Obtained with Ultrasonic–Microwave-Assisted Extraction
Source: Molecules. 2024 Feb 1;29(3):684. doi: 10.3390/molecules29030684 (PMC10856557; doi:10.3390/molecules29030684)
Supplement: Supplementary file 1 [file molecules-29-00684-s001.zip › molecules-2839502-supplementary.pdf]

Supporting Information

ABTS radical scavenging activity assay

Briefly, 2 mL of samples at different concentrations (0.5-2.5 mg/mL) were mixed evenly with 2 mL ABTS solution in the test tube. After incubation in dark for 6 min, the absorbance was measured at 734 nm as A_i. The control was determined by using distilled water instead of sample solution (A_0). ABTS radical scavenging activity was calculated according to the following formula:

$$\text{ABTS radical scavenging activity (\%)} = \left(1-\frac{A_i}{A_0}\right)\times 100 \tag{S1}$$

Hydroxyl radical scavenging activity assay

Briefly, 200 μL of samples at different concentrations (0.5-2.5 mg/mL) were mixed thoroughly with 5 mL ferrous (2 mmol/L) sulfate solution and 5 mL hydrogen peroxide solution (6 mmol/L). Then 2 mL salicylic acid solution (6 mmol/L) was added, and the absorbance was determined at 510 nm immediately after the mixture was evenly mixed, denoted as A_0. Distilled water was used to replace the sample for the blank control (A_1). The hydroxyl radical scavenging activity was calculated by the following equation:

$$\text{Hydroxyl radical scavenging activity (\%)} = \frac{A_0-A_1}{A_0}\times 100 \tag{S2}$$

Superoxide radical scavenging activity

1 mL of samples (0.5-2.5 mg/mL) were mixed evenly with 5 mL of Tris-HCl buffer solution (50 mmol/L, pH 8.2), then the mixture was placed in a water bath at 35 ℃ for 20 min. Then 1 mL of pyrogallol (10 mmol/L) was added and reacted for 4 min at 37 ℃. Afterwards, a drop of

concentrated HCl was added to terminate the reaction. Then the absorbance was determined at 320 nm as A_1 and the absorbance of Tris-HCl buffer and pyrogallic acid solution as A_0. The Superoxide radical scavenging activity was calculated as follows:

$$\text{Superoxide radical scavenging activity (\%)} = \frac{A_0\text{-}A_1}{A_0} \times 100 \quad \text{(S3)}$$
